# CDK1-Mediated Phosphorylation of BAG3 Promotes Mitotic Cell Shape Remodeling and the Molecular Assembly of Mitotic p62 Bodies

**DOI:** 10.3390/cells10102638

**Published:** 2021-10-02

**Authors:** Carole Luthold, Herman Lambert, Solenn M. Guilbert, Marc-Antoine Rodrigue, Margit Fuchs, Alice-Anaïs Varlet, Amélie Fradet-Turcotte, Josée N. Lavoie

**Affiliations:** 1 Centre de Recherche sur le Cancer, Université Laval, Quebec, QC G1R 3S3, Canada; carole.luthold@crchudequebec.ulaval.ca (C.L.); hermanlambert007@gmail.com (H.L.); solenn.guilbert@gmail.com (S.M.G.); r.marc.antoine92@gmail.com (M.-A.R.); margit.fuchs.cisssmc16@ssss.gouv.qc.ca (M.F.); aav57@cornell.edu (A.-A.V.); Amelie.Fradet-Turcotte@crchudequebec.ulaval.ca (A.F.-T.); 2 Oncology, Centre de Recherche du CHU de Québec-Université Laval, Hôtel-Dieu de Québec, Quebec, QC G1R 3S3, Canada; 3 Département de Biologie Moléculaire, Biochimie Médicale et Pathologie, Faculté de Médecine, Université Laval, Quebec, QC G1V0A6, Canada

**Keywords:** BAG3 1, HSPB8 2, p62/SQSTM1 bodies 3, CDK1 4, spindle positioning 5, mitotic cell rounding 6, actin 7, K63 polyubiquitin chain 8, protein quality control 9

## Abstract

The cochaperone BCL2-associated athanogene 3 (BAG3), in complex with the heat shock protein HSPB8, facilitates mitotic rounding, spindle orientation, and proper abscission of daughter cells. BAG3 and HSPB8 mitotic functions implicate the sequestosome p62/SQSTM1, suggesting a role for protein quality control. However, the interplay between this chaperone-assisted pathway and the mitotic machinery is not known. Here, we show that BAG3 phosphorylation at the conserved T285 is regulated by CDK1 and activates its function in mitotic cell shape remodeling. BAG3 phosphorylation exhibited a high dynamic at mitotic entry and both a non-phosphorylatable BAG3^T285A^ and a phosphomimetic BAG3^T285D^ protein were unable to correct the mitotic defects in BAG3-depleted HeLa cells. We also demonstrate that BAG3 phosphorylation, HSPB8, and CDK1 activity modulate the molecular assembly of p62/SQSTM1 into mitotic bodies containing K63 polyubiquitinated chains. These findings suggest the existence of a mitotically regulated spatial quality control mechanism for the fidelity of cell shape remodeling in highly dividing cells.

## 1. Introduction

During their progression through mitosis, cells undergo major and reversible changes in their shape and mechanics that contribute to accurate cell division. These cell shape changes depend on fine-tuning of multiprotein assemblies that are restricted to a narrow time-space framework [1,2]. Evidence indicates that protein quality control (PQC) systems, involving molecular chaperones of the heat shock protein (HSP) family and their cochaperones, contribute to the fidelity of cell division [3,4,5,6,7,8,9,10,11]. These PQC systems can assist the spatial and timely assembly–disassembly of protein complexes and resolve damages in proteins and cytoskeletal structures [12,13,14,15]. Indeed, recent findings support a role for PQC factors in the regulation of dynamic actin structures during mitosis [6,16,17,18,19]. Elucidating PQC mechanisms is therapeutically relevant since malignant tumor cells widely upregulate PQC systems and experience high mitotic stress [20,21].

BCL2-associated athanogene 3 (BAG3) is one of a family of cochaperones characterized by a BAG domain that regulates the fate of HSP70 substrates [22,23]. BAG3 is expressed in certain normal cell types but is upregulated in several cancer cell types [24]. BAG3 is unique for its association with the ATP-independent chaperones HSPB [25,26]. HSPB proteins bind damaged proteins and promote their sequestration in a native-like conformation to facilitate their subsequent repair or deposit to protective sites within the cell [25,26,27]. HSPB chaperones also play roles in maintaining cytoskeletal structure integrity [28,29]. BAG3 preferentially interacts with HSPB8, a divergent HSPB with a substrate-selective chaperone activity [30,31]. BAG3 also interacts with multiple signaling pathways via its modular protein structure and thereby acts as a signaling scaffold [24,32,33]. It is proposed that BAG3 serves as a molecular platform connecting chaperone systems to cellular pathways of protein degradation, nutrient sensing, cytoskeletal remodeling, and adaptation to proteotoxic stress and mechanical strain [34,35,36,37]. Mechanistically, the ability of BAG3 to regulate the sequestering activity of p62/SQSTM1 (hereafter referred to as p62) may play an important role, as p62 is a key molecule for proteostasis that has signaling functions [34,38,39]. In addition, BAG3 undergoes extensive post-translational modifications that may coordinate its multifaceted functions. For instance, site-specific BAG3 phosphorylation regulates the invasion of thyroid cancer cells [40], the aggresomal targeting of denatured protein [37], and the adaptive cell response to oxidative stress [41]. However, most of these post-translational modifications remain to be analyzed at the molecular and functional levels.

Recently, we described a novel mitotic function for BAG3 and HSPB8 in the timely remodeling of cell shape and actin-based structures that guide spindle orientation [6,42]. Cells depleted of BAG3 or HSPB8 show impaired mitotic cell rounding and are delayed at the prometaphase and metaphase stages. They eventually progress through anaphase, showing an increased incidence of aberrant cell division [6]. Actin polymerization inhibitors can normalize the mitotic phenotypes in BAG3-or HSPB8-depleted cells, suggesting a role for the chaperone complex in tuning actin assembly [18,19]. In line with this model, BAG3 depletion hinders the dynamic between the deacetylase HDAC6 and its substrate cortactin that possesses an acetylation-regulated actin-binding activity [18,43]. Moreover, the expression of an acetylation-mimic cortactin protein in BAG3-depleted cells can normalize mitotic cell rounding and the subcortical actin cloud organization that regulates spindle positioning [44,45]. Intriguingly, depletion of BAG3, HSPB8, and p62 can recapitulate the same mitotic phenotypes, arguing for a role of a PQC mechanism [6,18]. While BAG3 is hyperphosphorylated during mitosis, the functional significance of this modification for BAG3 mitotic phenotypes remains to be determined [6].

In this context, we hypothesized that site-specific BAG3 phosphorylation is required to activate its function in mitotic cell shape remodeling. In this study, we analyzed BAG3 mitotic phosphorylation at molecular and functional levels. We demonstrated that the mitotic kinase CDK1 phosphorylates T285 on BAG3 at the mitotic entry to facilitate the timed mitotic rounding and spindle orientation. Furthermore, we found that the BAG3 phosphorylation state and HSPB8 regulate the molecular assembly of p62 into dynamic mitotic bodies. We propose that the mitotic regulation of the BAG3-HSPB8-p62 signaling axis enables the cell to sequester superfluous cellular components or spatially segregate mitotic signaling, thereby assisting the rapid remodeling of cellular architecture.

## 2. Materials and Methods

### 2.1. Expression Vectors, Recombinant Adenoviruses, and Baculoviruses

BAG3-GFP constructs were generated by PCR using the BAG3-GFP^WT^ as template and the following primers:

BAG3^S284-T285A^.gen: 5′-CCA GCC AGG AGC GCC GCG CCA CTC CAC TCC-3′;
BAG3^S284-T285A^.rev: 5′-GGA GTG GAG TGG CGC GGC GCT CCT GGC TGG-3′;
BAG3^T285D^.gen: 5′-CCA GCC AGG AGC AGCGAC CCA CTC CAC TCC-3′;
BAG3^T285D^.rev: 5′-GGA GTG GAG TGG GTC GCT GCT CCT GGC TGG-3′;
BAG3^S289A^.gen: 5′-CGCCACTCCACGCCCCCTCGCCCATC-3′;
BAG3^S289A^.rev: 5′-GATGGGCGAGGGGGCGTGGAGTGGCG-3′;
BAG3^S291A^.gen: 5′-CTCCACTCCCCCGCGCCCATCCGTGTG-3′;
BAG3^S291A^.gen: 5′-CACACGGATGGGCGCGGGGGAGTGGAG-3′.

All constructs were confirmed by DNA sequence analyses. Recombinant adenoviruses for expression of BAG3-GFP proteins (WT, ΔPXXP [Δ302–418], and IPV [I96 V/V98 G/I208 G/V210 G]) were previously described [6]. Ad-BAG3-GFP^S284A-T285A^ (DMB), Ad-BAG3-GFP^S284A-T285A-S289A-S291A^ (QMB), and Ad-BAG3-GFP^T285D^ were produced by Welgen (Worcester, MA, USA), by subcloning the BAG3-GFP constructs into Adenovirus type C strain 5 vector lacking the E1 and E3 regions. All recombinant Ads were confirmed by sequencing of the inserted sequences. Recombinants Ads were amplified in the HEK293 VR cell line (a gift from Philip E. Branton, McGill University, Montreal, Quebec, Canada). Virus titers were determined using the AdenoX Rapid titer kit ClontechLaboratories (San Jose, CA, USA, #631028,). The CellLightBacMam 2.0 reagent (BacMam-RFP-α-tubulin) was purchased from Thermo Fisher Scientific (C10614, Waltham, MA, USA). pcDNA5/FRT/TO/BAG3-GFP constructs were generated by subcloning the BAG3-GFP cDNAs into pcDNA5/FRT/TO at the BamHI/XhoI fragment. pcDNA5/FRT/TO and pOG44 plasmids were from Thermo Fisher Scientific.

### 2.2. Antibodies and Chemicals

The following antibodies were used for immunoblotting (IB), immunofluorescence (IF), and immunoprecipitation (IP): rabbit BAG3 LP11 antibody (1:10,000 for IB) [6]. The phospho-specific pT285-BAG3 antibody was produced in rabbits. The antigen for rabbit immunization was prepared with the phospho-peptide CGSPARSS(phosT)PLHS crosslinked on Keyhole Lympets Hemocyanin (KLH) with dimethylformamide (MBS). Serum was affinity-purified using a 2-steps affinity/depletion purification designed for phospho-specific antibody and the Sulfolink Immobilization kit for peptides (Pierce, #44999). The resulting antiserum was concentrated and kept at −20 °C in 50% glycerol in PBS; pT285-BAG3 was used at 1:1000 for IB. For IB: Cyclin B1 (sc-245; 1:1000), GFP (sc-9996; 1:1000), p62 (sc-28359, 1:200), and CDK1 (sc-954; 1:1000) were from Santa Cruz Biotechnology (Dallas, TX, USA); GAPDH Clone 6 C5 (Fitzgerald, #10 R-G109 a; 1:10,000) and anti-Ub-K63 (#05–1308; 1:1000) were from Millipore (Oakville, ON, Canada); vinculin (V9131; 1:1000) was from Sigma-Aldrich (St Louis, MO, USA); p62 (#5114 S, 1:1000), pT269-S272-p62 (#; 1:1000), and pY15-CDK1 (#9111 S; 1:1000) were from Cell Signaling Technologies (Whitby, ON, Canada). For IP: GFP (A-11120; Molecular Probes/Thermo Fisher, Ottawa, ON, Canada); p62 (sc-13121; Santa Cruz Biothechnology). For IF: p62 (sc-28359; 1:50) was from Santa Cruz Biotechnology; α-tubulin (T5168; 1:1000) was from Abcam; α-tubulin (T5168; 1:1000) and Ub-K63 (#05–1308; 1:100) were from Millipore; pT269-S272-p62 (#13121; 1:200) was from Cell Signaling Technologies.

Hoechst Bisbenzimide H (B2261) and Alexa-Fluor 488 Phalloidin (A12379) were from Molecular Probes/Thermo Fisher; Fibronectin (10 μg/mL; F1141), Poly-L-Lysine (1 mg/mL; P1399) and Purvalanol A (10 µM, P4484) were from Sigma-Aldrich. RO-3306 (4 to 8 µM, SML0569), thymidine (2 mM, T9250), MG132 (5µM, Z-leu-leu-leu-al, C2211), doxycycline (1 to 10 ng/mL, D9891), were from Millipore Sigma. Nocodazole (100 to 400 ng/mL, #487928) and blasticidin (10 mg/mL; #203350) were from Calbiochem (San Diego, CA, USA). MLN8237 (1 µM, #1028486-01-2) was from Cedarlane (Burlington, ON, Canada). BI2536 (5 µM, #2 CT/09) was from JS Research Chemicals Trading (Wedel, Germany).

### 2.3. Cell Lines, Cell Culture, and Cell Synchronization

HeLa cells (ATCC) [46] and HeLa-RFP-H2B cells (a gift from Dr. S. Elowe, CR-CHU de Québec-Université Laval, Quebec, Canada) [47] were maintained in α-minimum essential medium (α-MEM) with 10% fetal bovine serum (FBS). Flp-In T-REx HeLa cells (a gift of Dr. A. Desai, Ludwig Institute for cancer research, San Diego) containing Flp-IN recombination sites and an inducible tetracycline/doxycycline promotor were maintained in α-MEM with 10% FBS, 100 µg/mL phleomycin D1 (Zeocin) and 10 mg/mL blasticidin S [48]. C2 C12 cells (mouse myoblasts; gift from Guillaume Grenier, Faculty of Medicine, Department of Orthopedic Surgery, Sherbrooke, Quebec) were maintained in DMEM High Glucose supplemented with 10% FBS. This cell line has been widely used as a model for skeletal muscle development (differentiation medium: DMEM High Glucose supplemented with 2% horse serum) [42]. All cell lines were grown in a humidified atmosphere with 5% CO_2_ at 37 °C. Flp-In T-REx HeLa-BAG3-GFP cell lines and Flp-In T-REx HeLa-p62-RFP cell line were generated by transfecting Flp-In T-REx HeLa cells with pcDNA5/FRT/TO containing the different BAG3-GFP constructs, GFP as a control, or p62-RFP together with pOG44. Selection of isogenic cell lines was made according to the manufacturer′s protocol (Thermo Fisher Scientific, Waltham, MA, USA). Cells were synchronized in mitosis by a double thymidine block (2 mM thymidine) [6]. Thymidine/nocodazole block was performed by a 24 h-incubation period with 2 mM of thymidine followed by a 3 h-release period in fresh medium. Cells were then synchronized in early mitosis by a 16 h-treatment with nocodazole (100 to 400 ng/mL). Mitotic cell extracts were prepared from cells recovered mechanically by a mitotic shake-off. Cells were synchronized in G2/M by an 18 h- RO-3306 (8 μM) block.

### 2.4. In Vitro Kinase Assay

cDNA encoding human BAG3 was cloned in phase with GST in vector pGEX-4 T3. Proteins were expressed in BL21 codon + (RIL) (Stratagene, San Diego, CA, USA), lysed by sonication in PBS supplemented with 0.1% Triton X-100, Complete (Roche, Mississauga, ON, Canada) and 1 mM DTT, purified using Glutathione-Sepharose 4 B (Pharmacia), dialyzed and concentrated in PBS. Histone H1 (H1917; Sigma-Aldrich), GST-BAG3 or GST were incubated with 3.5 units of CDK1/cyclinB1 (P6020 S; New England Biolabs, Whitby, ON, Canada) in 20 µL of 50 mM TRIS-HCl pH 7.4, 200 µM [γ-^32^ P]-ATP (3 µCi/tube, from Perkin Elmer, Woodbridge, ON, Canada), 10 mM MgCl_2_, and 2 mM DTT during 30 min at 30 °C. Proteins were separated on SDS-PAGE, stained with Coomassie BlueR250, and the radioactivity was revealed using FLA-5100 imaging system (Fujifilm Life Science). For cold in vitro kinase assay, 1 µg of GST-BAG3 or GST were incubated with 3 or 9 units of CDK1/cyclinB1 (P6020 S; New England Biolabs) in 20 µL of 15 mM TRIS-HCl pH 7.4, 250 µM ATP, 12.5 mM MgCl_2_, and 0.6 mM DTT during 45 min at 30 °C.

### 2.5. Knockdown-Rescue Experiments, RNA interference, and Biochemistry

For knockdown experiments, HeLa cell lines were transfected with 50 nM or 75 nM short interfering RNA (siRNA) duplexes for 16 h by the calcium phosphate transfection method [42] or using Lipofectamine RNAiMax Reagent (Thermo Fisher Scientific, Waltham, MA, USA), following the manufacturer′s recommendations [42]. The siRNA duplexes were based on human sequences and were purchased from Qiagen (HPP grade siRNA, Montreal, QC, Canada) or Thermo Fisher Scientific (standard A4 grade), and control siRNA (siCtl) AllStars Negative Control was purchased from Qiagen. Sequences of the sense strands are as follows:
siCtl allStars5′-CAGGGTATCGACGATTACAAA-3′siBAG3 (ORF)5′-CGAAGAGTATTTGACCAAA-3′; formerly described as siBAG3_1 [6]siBAG3(3′UTR_1)5′-GATGTGTGCTTTAGGGAAT-3′; formerly described as siBAG3_3 [6]siBAG3(3′UTR_2)5′-CTGACTTTAGAGAGAGTAATT-3′siHSPB8_15′-CAGAGGAGTTGATGGTGAA-3′; formerly described as siHSPB8_3 [6]siHSPB8_25′-GCAGTGAATGCAAGGGTTATT-3′ [6]sip62_15′-GGAAATGGGTCCACCAGGA-3′ [6]sip62_25′-AGACCAAGAACTATGACAT-3′ [6]

For depletion-rescue experiments, we used a protocol based on the adenofection method, as described [42]. Briefly, HeLa-RFP-H2B cells were transduced with Ad-BAG3-GFP viruses using 1 plaque-forming unit per cell (pfu/cell) together with BacMam 2.0 reagents (RFP-α-tubulin) at 4 pfu/cell, and simultaneously transfected with siRNA directed to BAG3 3′-UTR (siBAG3 [3′UTR_1] or siBAG3 [3′UTR_2]) during 48 h; cells were synchronized by a double thymidine block (2 mM). For preparing BAG3-GFP IPs, asynchronous HeLa cells or cells arrested in mitosis by a 16 h-treatment with nocodazole (200 to 400 ng/mL) were washed in ice-cold PBS and were lysed by 3 cycles of freezing/thawing in 4-volume of lysis buffer (20 mM TRIS-HCl pH 7.6, 150 mM NaCl, 1 mM EDTA, 0,1% IGEPAL, 1 mMNaVO_4_, 10 mM NaF, 40 mM β-glycerophosphate, 1 × Complete [Roche], 1 mM DTT). Cell extracts were centrifuged at 15,000× *g* for 15 min, and equal amounts of supernatants were incubated with specific antibodies that have been coupled to protein A on Dynabeads (Life Technologies, Carlsbad, CA, USA) for 60 min. Immune complexes were then collected on a magnetic stand and washed 3 times in lysis buffer. Equal amounts of immune complexes were loaded on SDS-PAGE and analyzed by Western blot as described [49]. Protein concentrations were determined with the DC protein assay reagent (Bio-Rad Laboratories, Hercules, CA, USA) and densitometric analyses were performed with FluorS MAX MultiImager-captured images using the QuantityOne software version 4.5.0 (Bio-Rad Laboratories). HeLa Flp-in T-Rex cell lines were treated with 1 ng/mL of doxycycline for 16 h to induce BAG3-GFP protein expression.

### 2.6. Immunofluorescence, Microscopy, and Live-Cell Imaging

For immunostaining of mitotic spindle and insoluble mitotic structures, cells were fixed in 4% formaldehyde in 0.2% Triton X-100, 20 mM Pipes, pH 6.8, 1 mM MgCl2, 10 mM EGTA, pH 8.0, 4% formaldehyde for 10 min at room temperature (RT); alternatively, cells were fixed with 4% paraformaldehyde for 15 min at RT or in cold methanol (staining of whole p62 structures). Specimens were then processed for immunofluorescence [50,51], using the indicated primary antibodies and Alexa Fluor goat anti-rabbit or goat anti-mouse antibodies (Molecular Probes/Thermo Fisher Scientific, Waltham, MA, USA). DNA was stained with cell-permeable Hoechst and F-actin was stained with Alexa Fluor phalloidin (Molecular Probes/Thermo Fisher Scientific). Phenotypes were monitored routinely by at least two independent investigators by visual inspection of fixed specimens and were scored in a blind manner. Phenotypic analyses and epifluorescence images were performed with an AxioObserver Z1 system using a 40 × Plan-Neofluoar 0.6 NA objective, and a charged-coupled device (CCD) camera AxiocamMRm controlled by the Zen 2 software version 2.0.14283.302 (Zeiss, Toronto, ON, Canada). Mitotic rounding was quantified by visual inspection of defects in mitosis as follows: partially rounded or flat mitotic cells. Defects in aggresome formation were scored by the presence of microaggregates versus a perinuclear aggresome defined as a cluster of ubiquitinated proteins localized close to the nucleus. Confocal microscopy of fixed cells for MIBS quantification, and live-cell imaging were performed with a Perkin Elmer UltraVIEW Spinning Disk Confocal (60 × oil 1.4 NA or 40 × 0.75 NA objectives, respectively) equipped with an EMCCD cooled charge-coupled camera at −50 °C (Hamamatsu Photonics K.K) and driven by Volocity software version 6.01, equipped with a humidified 5% CO_2_ thermoregulated chamber (Nikon, Melville, NY, USA). For live-cell imaging, HeLa-RFP-H2B cells were seeded on fibronectin-coated glass dishes (MatTek Corporation, Ashland, MA, USA) and images were acquired starting at 48 h post-transfection, at 90-s intervals for 75 min. Mitotic phenotypes were estimated by visual inspection of time sequences. Cells expressing an upper threshold level of BAG3 constructs were excluded from quantitative analyses. BAG3-dependent mitotic defects were scored considering spindle rotation (rocking) or mispositioning and/or delay in mitosis and were estimated from individual cells from 3 independent experiments. MIBS number per cell were quantified from confocal image stacks using the Volocity software based on the intensity and size of objects using a minimum threshold of 0.5 µm; alternatively, the MIBS number and size were scored from confocal image stacks, by using the 3D imager of the NIS Elements AR software version 5.02.00 (Build 1266) Patch 02 (Nikon). The structures were selected through a minimum intensity threshold and a minimum size of 0.5 µm. Confocal microscopy of fixed cells for MIBS containing pS403-p62 or pT269-S272-p62 was performed with an AxioObserver Z1 system Zeiss LSM 700 (Plan-Apochromat 63 × 1.40 NA oil objective) and driven by Zen 2009 (Zeiss).

### 2.7. Statistical Analyses

Statistical analyses were performed using the Prism 9.0 software version 9.2.0 (GraphPad Software). For experiments examining the proportions of cells with mitotic defects or rounding defects in depletion-rescue experiments, the number of cells with defects was analyzed relative to the total number for each condition using the Fisher’s exact test, which is used for small sample sizes with nominal/categorical variables. For experiments examining MIBS assembly, the numbers of MIBS per cell were quantified for each condition and statistically analyzed using the Kruskal–Wallis test (combined with Dunn′s multiple comparisons), which is a nonparametric test that compares more than two unpaired groups of ordinal or numerical variables.

## 3. Results

### 3.1. BAG3 Is Phosphorylated at T285 at Mitotic Entry

**Figure 1:**
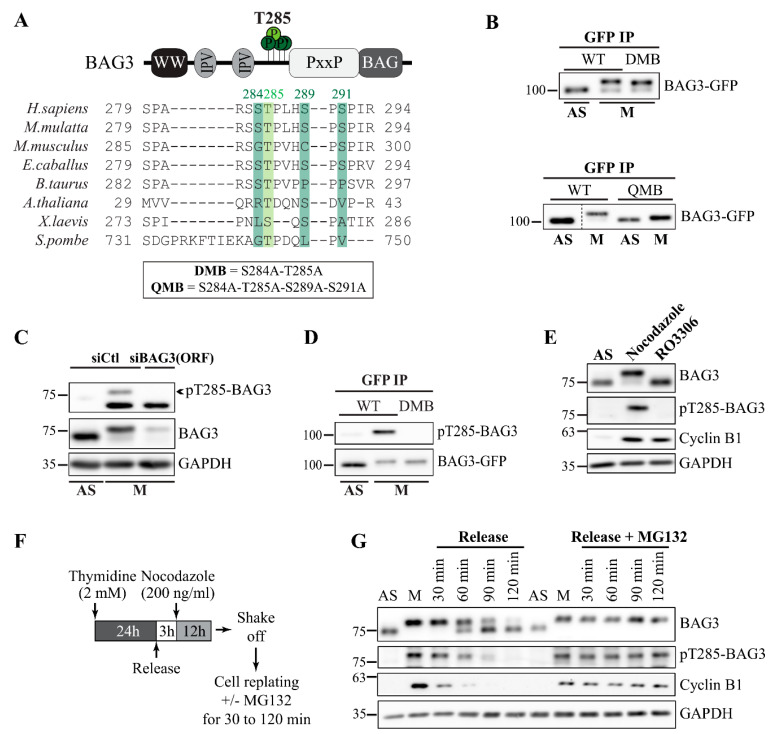
BAG3 is phosphorylated at T285 during mitosis. (**A**) Schematic representation of BAG3 modular domains and phosphorylated residues, as identified by AP-MS. See also Figure S1 for MS data. Sequence alignments show the interspecies conservation of the identified phosphorylated motif. BAG3-GFP^DMB^ and BAG3-GFP^QMB^ constructs were generated by alanine substitution of designated residues (box). (**B**) Western blots of BAG3-GFP IPs prepared from HeLa-RFP-H2B cells growing asynchronously (AS) or arrested in mitosis by nocodazole treatment (M) and recovered by a mitotic shake-off, showing the mitotic mobility shift of BAG3-GFP constructs. Cells were transfected with BAG3-specific siRNA and transduced with Ad-BAG3-GFP. (**C**) Representative Western blots of HeLa cell extracts that have been transfected with control siRNA or BAG3-specific siRNA targeting BAG3 open reading frame (siBAG3 [ORF]), showing the pT285-BAG3 antibody specificity; M: nocodazole-arrested cells recovered by a mitotic shake-off; AS: asynchronous cells; levels of total BAG3 and GAPDH (loading control) are shown. (**D**) BAG3-GFP IPs were prepared from asynchronous (AS) or from mitotic HeLa-RFP-H2B cells synchronized by nocodazole and recovered by mitotic shake-off after transduction of cells with Ad-GFP-BAG3, as indicated; Western blots show total levels of GFP-BAG3 proteins and pT285-BAG3. (**E**) Extracts were prepared from asynchronous (AS) or from mitotic HeLa-RFP-H2B cells synchronized in early mitosis by nocodazole or arrested at the G2/M stage by RO3306. Western blots of endogenous proteins were performed as indicated; GAPDH: loading control. (**F**) Schematic of the protocol used. HeLa-RFP-H2B cells synchronized in mitosis by nocodazole, were recovered by a mitotic shake-off, and then seeded on poly-L-lysine-coated dishes in fresh medium, with or without MG132 (5 µM) to inhibit mitotic exit. (**G**) Cells were collected at different times and extracts were analyzed by Western blots, as indicated; GAPDH: loading control.

BAG3 is hyperphosphorylated at mitotic entry and exhibits a supershifted band on SDS-PAGE [6]. To address the functional relevance of BAG3 phosphorylation, we first sought to identify BAG3 residues that are phosphorylated during mitosis. We performed affinity purification (AP) combined with mass spectrometry (MS) in HEK 293T cells that have been synchronized in mitosis by treatment with nocodazole, a microtubule poison arresting the cells in early mitosis. In-gel digestion of the BAG3 supershifted band reproducibly identified four phospho-residues: pS289, pS291, pS284, and pT285 with good confidence of phosphorylation site-specific localization (Figure S1A, M: red box; Figure S1B; see maximum Ascore for each site in Figure S1C–F) [52]. These four residues were reported to be phosphorylated at high frequencies, according to the PhosphoSitePlus database [53]. Comparison of BAG3 sequences from different species revealed a high degree of conservation around these four residues. Moreover, these residues are located within a region lying between the IPV and PXXP motifs that both regulate BAG3 mitotic phenotypes (Figure 1A) [6]. In-gel digestion of the lower BAG3 band from asynchronous cells further revealed that S289 and S291 were also phosphorylated during interphase (Figure S1A, AS: blue box; Figure S1B). However, neither pT285 nor pS284 were detected in the BAG3 band from asynchronous cells (Figure S1B). These results suggest that the S284-T285 phospho-motif on BAG3 is mitotically regulated.

To determine the contribution of these phospho-residues to BAG3 mitotic mobility shift, we produced BAG3-GFP constructs bearing substitutions of both S284 and T285 to non-phosphorylatable alanine (BAG3-GFP^DMB^), or substitutions of all four residues to alanine (BAG3-GFP^QMB^; Figure 1A). Since these residues are close to each other, we reasoned that the impact of a single residue substitution could be mitigated by compensatory phosphorylation on neighbor residues. BAG3-GFP proteins were expressed in HeLa cells by transduction of recombinant adenoviruses, immunopurified, and their mobility was analyzed on SDS-PAGE by immunoblotting with an anti-GFP antibody [6,42]. In mitotic cells recovered by a mitotic shake-off (M), wild-type BAG3-GFP mostly displayed a supershifted band (Figure 1B). Under these conditions, alanine substitution of S284 and T285 induced a modest reduction in BAG3 mobility shift (Figure 1B, DMB). A similar decrease was observed upon alanine substitution of either S289 or S291 (unpublished data). However, alanine substitution of all four sites strongly decreased BAG3 mobility shift in mitotic cells (Figure 1B, QMB). Thus, we concluded that these phospho-residues may all contribute to BAG3 hyperphosphorylation during mitosis.

We selected the S284-T285 phospho-motif for functional analyses, based on our AP-MS data indicating mitotic-specific phosphorylation on these residues (Figure S1B). To first validate BAG3 mitotic phosphorylation at this motif, we generated a phospho-specific antibody against a T285-phosphorylated peptide of BAG3 (CGSPARSS[pT]PLHS), as pT285 may be preferentially phosphorylated according to MS data (Figure S1B). The pT285-BAG3 antibody detected a mitotic-specific band in total cell lysates from HeLa cells, which was reduced upon transfection of cells with BAG3-specific siRNAs (Figure 1C; Figure S2A, siBAG3 [ORF]). Moreover, the pT285-BAG3 antibody recognized wild-type BAG3-GFP immunopurified from mitotic cells but did not react to a non-phosphorylatable BAG3-GFP^DMB^ protein, confirming its specificity for the pT285 site (Figure 1D, DMB).

We further detected pT285-BAG3 along with BAG3 supershifted band in HeLa cells arrested in mitosis by nocodazole treatment. However, this phospho-specific band was not detected in cells arrested at the G2/M stage by treatment with the CDK1 inhibitor RO3306 (Figure 1E). Moreover, after reversing nocodazole activity by a cell wash, pT285-BAG3 and BAG3 supershifted band intensity peaked in early mitosis and were gradually downregulated along with cyclin B1 levels, as cells progressed to anaphase (Figure 1F,G). However, when mitotic exit was inhibited by an MG132 treatment that blocks cyclin B1 degradation, high levels of pT285-BAG3 and the BAG3 supershifted band could be maintained (Figure 1G, release + MG132) [54]. These results indicate that BAG3 phosphorylation at T285 is specifically regulated by mitotic signaling.

### 3.2. BAG3 Phosphorylation at T285 Is Regulated by CDK1

**Figure 2:**
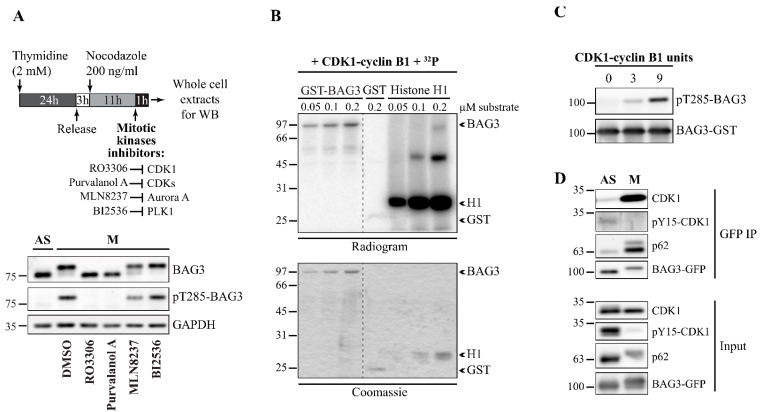
CDK1 regulates BAG3 phosphorylation dynamic at T285. (**A**) Schematic of the protocol used. Extracts were prepared from mitotic HeLa-RFP-H2B incubated for 1 h in the presence of chemical inhibitors of the following kinases: RO3306 (CDK1, 8 µM), Purvanolol A (CDKs, 10 µM), MLN8237 (Aurora A, 1 µM), and BI2536 (PLK1, 1 µM) or the vehicle only (DMSO); mitotic cells were collected by a mitotic shake-off. Immunoblotting was performed as indicated; GAPDH: loading control. See also Figure S2. (**B**) Autoradiogram showing phosphorylation of recombinant GST-BAG3 by a purified CDK1-cyclin B1 complex in the presence of ^32^ P-ATP; positive control: Histone H1; negative control: GST. (**C**) Immunoblotting of in vitro phosphorylated BAG3 using the phospho-specific pT285-BAG3 antibody; total levels of GST-BAG3 are shown. (**D**) BAG3-GFP IPs were prepared from asynchronous (AS) or mitotic HeLa-Flp-In T-REx- BAG3-GFP^WT^ (nocodazole-arrested) transfected with BAG3-specific siRNA (siBAG3 [3′UTR_1], 48 h) and treated with doxycycline to induce BAG3-GFP expression (1 ng/mL, 16 h). Western blots of the GFP IPs were performed as indicated; levels of CDK1, pY15-CDK1 (inactive CDK1), p62, and BAG3-GFP in total cell extracts are shown (Input).

Computational analyses revealed that S284 and T285 phosphosites on BAG3 are candidate target sites of CDK1. The same sites were identified as putative CDK1 substrates by quantitative phosphoproteomics analyses in HeLa cells [55]. To test the dependence of BAG3 mitotic phosphorylation on various mitotic kinases, HeLa-RFP-H2B cells were arrested at mitotic entry by a thymidine/nocodazole block to induce BAG3 phosphorylation and were then treated for an additional hour with pharmacological inhibitors of mitotic kinases, including RO3306 (CDK1), purvanolol A (pan-CDKs), MLN8237 (Aurora A), and BI2536 (PLK1) (see a schematic of the protocol, Figure 2A) [56,57,58]. We found that the CDK inhibitor purvanolol A and RO3306, a CDK1-specific inhibitor, abolished BAG3 mobility shift and its phosphorylation at T285 (Figure 2A). In contrast, Aurora A and PLK1 inhibitors only partially decreased or had no impact on BAG3 phosphorylation, respectively (Figure 2A). Shorter treatments with RO3306 also reduced the pT285-BAG3 signal along with cyclin B1 levels in thymidine/nocodazole-arrested cells (Figure S2B). To further assess a requirement for CDK1 activity, cyclin B1 degradation and mitotic exit were blocked by adding MG132. Under these conditions, BAG3 mobility shift was still partially inhibited (Figure S2C). These results suggest that BAG3 mitotic phosphorylation is regulated by CDK1.

To then determine if BAG3 is a substrate of CDK1, we performed in vitro phosphorylation assays with radiolabeled [γ-^32^ P] using purified active CDK1-cyclin B1 complex and bacterially expressed GST-BAG3 as a substrate. We observed dose-dependent phosphorylation of GST-BAG3 along with the phosphorylation of the positive control, histone H1, but not of GST (Figure 2B). Moreover, immunoblotting of in vitro phosphorylated GST-BAG3 confirmed phosphorylation of BAG3 at T285 by CDK1 (Figure 2C). To monitor BAG3 interaction with CDK1 in cells, we performed coimmunoprecipitation assays in HeLa Flp-In T-REx cells expressing BAG3-GFP. We detected a clear association between BAG3-GFP and the activated form of CDK1 (not phosphorylated on the inhibitory Y15) in mitotic cells (M) but not in asynchronous cells (AS) (Figure 2D). As shown before, we also detected an increased BAG3 association with p62 that also displayed a mitotic mobility shift (Figure 2D) [6,59]. These results indicate that BAG3 is a CDK1 substrate and identify T285 as a CDK1-regulated phosphosite during mitosis.

### 3.3. BAG3 Phosphorylation Dynamics at S284-T285 Regulate Mitotic Cell Shape Remodeling

**Figure 3:**
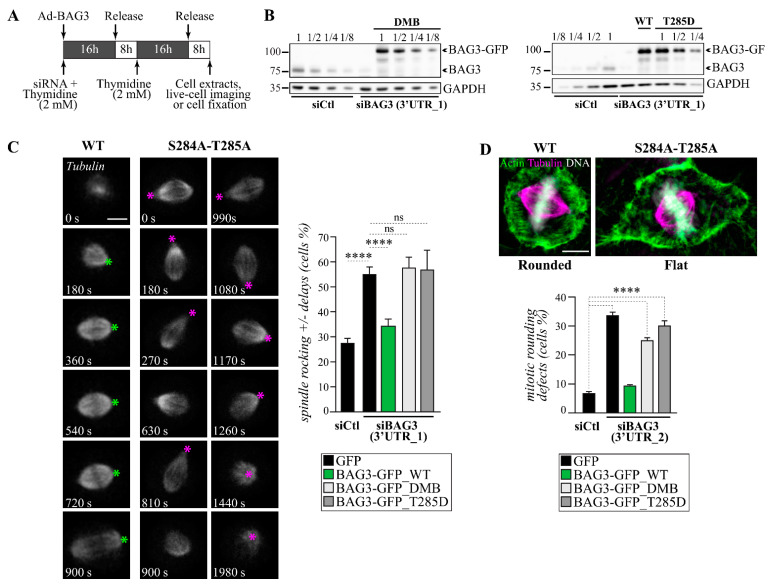
Non-phosphorylatable BAG3-GFP^DMB^ expression in BAG3-depleted cells, as phospho-mimetic BAG3-GFP^T285D^, cannot rescue spindle dynamics and mitotic rounding defects. (**A**) Depletion-rescue experiments were performed in HeLa-RFP-H2B transfected with siBAG3 (3’UTR_1) or control siRNA and transduced with recombinant adenoviruses driving expression of BAG3-GFP proteins. Cells were synchronized in mitosis by a double thymidine block. (**B**) Total cell extracts were analyzed by Western blots using anti-BAG3 and anti-GAPDH; levels of endogenous BAG3 depletion (>75% reduction) and exogenous BAG3-GFP levels were estimated by loading increasing amounts of control cell extract (siCtl, 1/8, 1/4, 1/2, 1), or from an extract of BAG3-depleted cells transduced with Ad-BAG3-GFP, respectively. (**C**) Representative spinning disk confocal time-lapse sequences of cells from (**B**) transduced with BacMam-RFP-α-tubulin, showing normalization of spindle dynamics in BAG3-depleted cells upon reintroduction of BAG3-GFP^WT^, but not by non-phosphorylatable BAG3-GFP^DMB^ expression or phosphomimetic BAG3-GFP^T285D^. The green asterisks designate a spindle pole showing normal dynamic in a BAG3-depleted cells expressing wild-type BAG3-GFP, while magenta asterisks designate a spindle pole showing abnormal motility in a BAG3-depleted cell expressing BAG3-GFP^DMB^; Bar: 10 μm. The graph shows quantification of percentages of rounded cells with spindle dynamic defects defined as spindle rocking or stalled in mitosis ± spindle rocking; means ± SE of 92 to 525 cells from at least 3 independent experiments. Statistical significance was analyzed with the Fisher Exact Test ****: *p* < 0.0001. See also Videos S1–S5 for representative phenotypes. (**D**) Epifluorescence images showing the representative phenotype of a rounded mitotic cell at metaphase (siCtl) compared to a flat mitotic cell at metaphase (siBAG3 [3′UTR_2]); F-actin, tubulin, and DNA were stained using phalloidin (green), α-tubulin antibody (magenta) and Hoechst (white), Bar: 10 μm. The graph depicts percentages of cells with mitotic cell rounding defects; means ± SE of 600 to 622 cells from 3 independent experiments. Statistical significance was analyzed by the Fisher Exact Test ****: *p* < 0.0001; ns, not significant. See also Figure S2A for Western blots of BAG3 depletion levels and Figure S3 for impacts of BAG3-GFP constructs on the aggresomal targeting of ubiquitinated proteins.

We showed before that the depletion of either BAG3 or its mitotic partners, HSPB8 and p62, can recapitulate the same defects in spindle dynamics in rounded mitotic cells [6,18]. Moreover, a significant proportion of siRNA-treated cells are unable to round up at onset of mitosis. Both phenotypes were linked to a misregulation of mitotic actin remodeling [6,18]. Thus, we next sought to determine the impact of BAG3 phosphorylation on relevant mitotic phenotypes associated with the BAG3-HSPB8-p62 signaling axis. To this end, we performed depletion-rescue experiments using wild-type BAG3-GFP^WT^ and BAG3-GFP proteins bearing substitutions of S284 and T285 to a non-phosphorylatable alanine (BAG3-GFP^DMB^). Although T285 may be the preferred phospho-site, we mutated S284 to avoid compensatory phosphorylation in the absence of T285. We also substituted T285 to a phosphomimetic aspartic acid (BAG3-GFP^T285D^). Using recombinant adenoviruses, BAG3-GFP proteins were expressed at similar levels in HeLa-RFP-H2B cells depleted of endogenous BAG3 by transfection with siRNA duplex targeting the 3′ untranslated region of BAG3 (Figure 3A,B; Figure S2A) [42]. We first determined the impact of BAG3-GFP proteins on spindle dynamics by live-cell imaging of rounded mitotic cells following expression of low levels of RFP-α-tubulin. In agreement with previous findings, BAG3 depletion increased the level of spindle orientation defects that was restored near to the control cell level by the expression of BAG3-GFP^WT^ but not GFP (Figure 3C; Videos S1–S3) [6]. This BAG3 phenotype was typified by unusually motile mitotic spindles (spindle rocking, as designated by asterisks in Figure 3C and Video S2) and was often associated with a mitotic delay, in agreement with our previous findings [6]. In contrast, neither non-phosphorylatable BAG3-GFP^DMB^ expression in BAG3-depleted cells, nor phosphomimetic BAG3-GFP^T285D^ could restore accurate spindle dynamics (Figure 3B,C; Videos S4 and S5). We then analyzed the effect of BAG3-GFP proteins on mitotic cell rounding after staining of actin filaments [6,18]. As expected, there was a significant increase in the proportion of BAG3-depleted cells that failed to round up at mitotic entry relative to cells transfected with a control siRNA (Figure 3D; Figure S2A). This BAG3 phenotype was corrected by BAG3-GFP^WT^ expression but not by the non-phosphorylatable BAG3-GFP^DMB^ or phosphomimetic BAG3-GFP^T285D^ (Figure 3D). These results suggest that a dynamic phosphorylation at T285 on BAG3 regulates mitotic cell shape remodeling.

To further assess the functionality of BAG3-GFP proteins, we analyzed their ability to sustain BAG3 function during stress, in the aggresomal targeting of ubiquitinated proteins. Cells were treated with the proteasome inhibitor MG132 and aggresome formation was monitored microscopically after ubiquitin staining. As shown before, BAG3 depletion impaired aggresome formation that could be restored by BAG3-GFP^WT^ expression (Figure S3) [34,35,37]. Remarkably, non-phosphorylatable BAG3-GFP^DMB^ expression could also restore aggresome formation in BAG3-depleted cells (Figure S3). Moreover, both the non-phosphorylatable BAG3-GFP^DMB^ and phosphomimetic BAG3-GFP^T285D^, as wild-type BAG3-GFP, were able to alleviate the reduction in HSPB8 levels in BAG3-depleted (Figure 7B) [6,19]. These results suggest that while BAG3-GFP^DMB^ and BAG3-GFP^T285D^ constructs are unable to sustain accurate BAG3 function during mitosis, they still retain some BAG3-specific functions.

### 3.4. BAG3 Mitotic Phosphorylation Is Regulated by p62

Having established that BAG3 mitotic phosphorylation has functional implications, we then sought to explore the regulatory mechanism. We showed that associations between BAG3, HSPB8, and p62 are increased at mitotic entry, suggesting that they form a ternary complex [6]. Since p62 directly interacts with CDK1 to regulate its activation during mitosis, we reasoned that p62 could regulate BAG3 phosphorylation [59]. In line with this hypothesis, coimmunoprecipitation assays in mitotic HeLa cells suggested that BAG3 associations with CDK1 and p62 depend on the same structural requirements: the HSPB8 binding motif (IPV) and the PXXP domain on BAG3, which are both required for BAG3 mitotic function (Figure S4A) [6].

**Figure 4:**
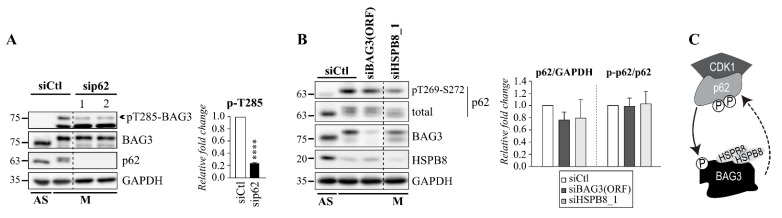
BAG3 phosphorylation at T285 is regulated by p62 during mitosis. (**A**) Western blots of extracts from asynchronous (AS) or mitotic HeLa cells (M) synchronized in mitosis by nocodazole and collected by mitotic shake-off. Cells were transfected with control siRNA (siCtl) or p62-specific siRNAs (sip62) and levels of pT285-BAG3, BAG3, p62, and GAPDH (loading control) are shown. Graph depicting the fold change in pT285-BAG3 levels, as estimated relative to cells transfected with control siRNA and normalized relative to GAPDH levels; means ± SE from 3 independent experiments. Statistical significance was analyzed by the Student′s *t*-Test ****: *p* < 0.0001. See also Figure S4B that depicts the fold change in BAG3 total levels as estimated relative to cells transfected with control siRNA and normalized relative to GAPDH levels. (**B**) Western blots of cell extracts prepared from asynchronous (AS) or from mitotic HeLa cells (M) synchronized in mitosis by nocodazole and collected by mitotic shake-off; cells were transfected with control siRNA (siCtl) or BAG3-specific siRNA (siBAG3 [ORF]) or HSPB8-specific siRNAs, and protein levels are shown as indicated. Graphs depicting the fold change in total p62 levels (p62) normalized relative to GAPDH levels, and the fold change in pT269-S272-p62 levels (p-p62) normalized relative p62 total levels, as estimated relative to cells transfected with control siRNA. Data are means ± SE from 3 independent experiments. (**C**) Scheme recapitulating putative functional relationships: CDK1 phosphorylates p62 that in turn enhances CDK1 activity [59]; the increased mitotic association between the BAG3-HSPB8 chaperone complex and p62 may promote BAG3 phosphorylation and stabilize p62 during mitosis.

To examine the impact of p62 on BAG3 mitotic phosphorylation, HeLa cells were treated with p62-specific siRNAs and synchronized in mitosis. The levels of pT285-BAG3 were then analyzed by immunoblotting with pT285-BAG3 antibody. Remarkably, p62 depletion significantly reduced BAG3 phosphorylation at T285 without significantly affecting BAG3 total levels (Figure 4A; Figure S4B). Intriguingly, we observed that the depletion of BAG3 or HSPB8 tended to reduce p62 whole levels along with p62 phosphorylation at CDK1 target sites (T269-S272) (Figure 4B). Although not statistically significant, these effects were reproducible, suggesting that p62 levels may be stabilized by BAG3 and HSPB8 during mitosis. Together, the results suggest that the mitotic p62-CDK1 interaction combined with the recruitment of p62 by the BAG3-HSPB8 chaperone complex may facilitate BAG3 phosphorylation at mitotic entry (Figure 4C) [6,59].

### 3.5. BAG3 Phosphorylation and HSPB8 Regulate the Molecular Assembly of p62 Bodies during Mitosis

To further investigate the significance of the CDK1-regulated BAG3-HSPB8-p62 signaling axis, we analyzed p62 subcellular organization during mitotic progression. The molecular assembly of p62 oligomers into higher-ordered inclusion bodies is a key process that determines p62 functions in protein sequestration, autophagosome assembly, and cytoprotective signaling [60,61,62,63,64,65,66,67]. However, p62 organization during mitosis remains unknown. Cellular inclusions are believed crucial to regulate spatial PQC, by segregating and sequestering damaged or superfluous proteins. They also provide dynamic platforms to coordinate cell signaling and PQC mechanisms during physiological processes [14,15,68]. We reasoned that the mitotic BAG3-HSPB8-p62 signaling axis could regulate p62 molecular assembly during mitotic progression.

**Figure 5:**
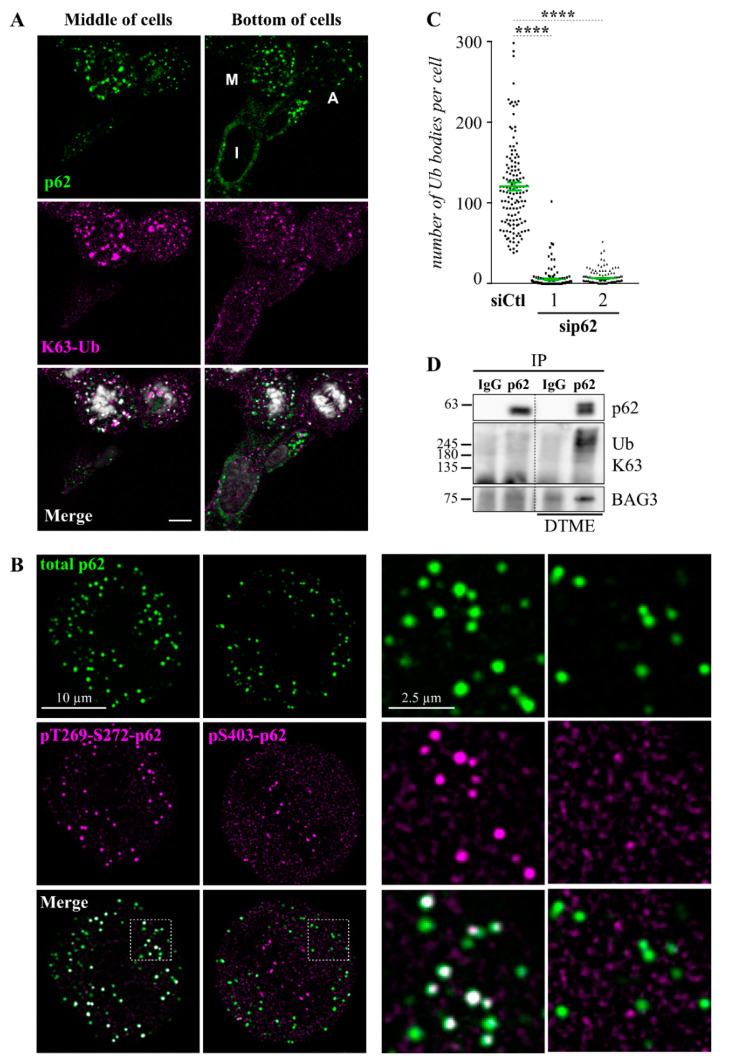
The sequestosome p62 forms mitotic inclusion bodies (MIBS) enriched in CDK1-phosphorylated p62 and K63 polyubiquitin chains. (**A**) Representative single-plane confocal images of HeLa cells synchronized in mitosis by a double thymidine block, showing staining of p62, K63 polyubiquitin (K63-Ub), and DNA (Hoechst) in cells at different mitotic stages compared to a cell in interphase; M: metaphase; A: anaphase; I: interphase. Bar: 10 μm. (**B**) Deconvolved single-plane confocal images of a representative HeLa cell at metaphase from a non-synchronized cell population, showing staining of pT269-S272-p62 (CDK1-induced), pS403-p62 (stress-induced), and total p62. Enlarged views of the boxed regions emphasize MIBS spherical shape and enrichment in p62 phosphorylated at mitotic sites (T269-S272) but not at the canonical stress-induced site (S403); Bars: 10 μm or 2.5 µm. (**C**) Quantification of polyubiquitin clusters in non-synchronized HeLa cells at prometaphase-metaphase. Cells were transfected with control siRNA or p62-specific siRNAs and stained using anti-α-tubulin and anti-ubiquitin antibodies, means ± SE from 102 to 136 cells from at least 4 independent experiments. Statistical significance was analyzed using the Kruskal–Wallis test and Dunn′s multiple comparisons: ****, *p* < 0.0001. See also Figure S5A for the representative phenotype in p62-depleted cells and Figure S5B for MIBS staining in other cell types. (**D**) p62 or control IPs were prepared using anti-p62 or rabbit IgG, respectively, from mitotic HeLa cells (nocodazole-treated and recovered by a mitotic shake-off); cells were treated with a reversible crosslinker (DTME) or the vehicle prior to cell lysis and p62 IPs were analyzed by Western blot using anti-p62, anti-BAG3, and anti-K63 polyubiquitin.

To determine this possibility, we first performed immunostaining of p62 and polyubiquitin chains, as the propensity of p62 clustering depends on polyubiquitin binding with a preference for K63-linked ubiquitin chains (K63-Ub) [69,70,71]. Confocal imaging of HeLa cells synchronized in mitosis by a thymidine block revealed that cells at different mitotic phases exhibited a marked increase in K63-Ub-positive p62 clusters relative to interphase cells (Figure 5A). These p62 mitotic clusters showed a spherical shape that was reminiscent of p62 protein condensates (Figure 5B, see the enlarged view of boxed regions) [72]. As expected, the number of mitotic polyubiquitin chain-positive p62 bodies was downregulated by p62 depletion (Figure 5C; Figure S5A). These p62 mitotic structures were also observed in non-synchronized cells populations from different proliferative cell types, including normal mouse myoblasts C2C12 cells, arguing for a mitotic-specific function (Figure S5B). Moreover, they were enriched in p62 phosphorylated at CDK1-target sites (pT269-S272-p62), but not in p62 phosphorylated at the canonical stress-induced site (pS403-p62), indicating that they differ from stress-induced p62 bodies (Figure 5B) [59,73]. Notably, when p62 was immunopurified from mitotic cells, we could barely detect p62-associated K63 polyubiquitin chains biochemically. However, the biochemical interaction between p62 and K63 polyubiquitin proteins were preserved in dithiobismaleimidoethane (DTME) cross-linked cells (Figure 5D). Importantly, BAG3 also showed an enhanced association with p62 in DTME cross-linked cells (Figure 5D). Together, these data suggest that p62 assembles into **m**itotic **i**nclusion **b**odie**s** (hereafter named MIBS) that may dynamically interact with BAG3 and K63 polyubiquitin chains-containing proteins.

**Figure 6:**
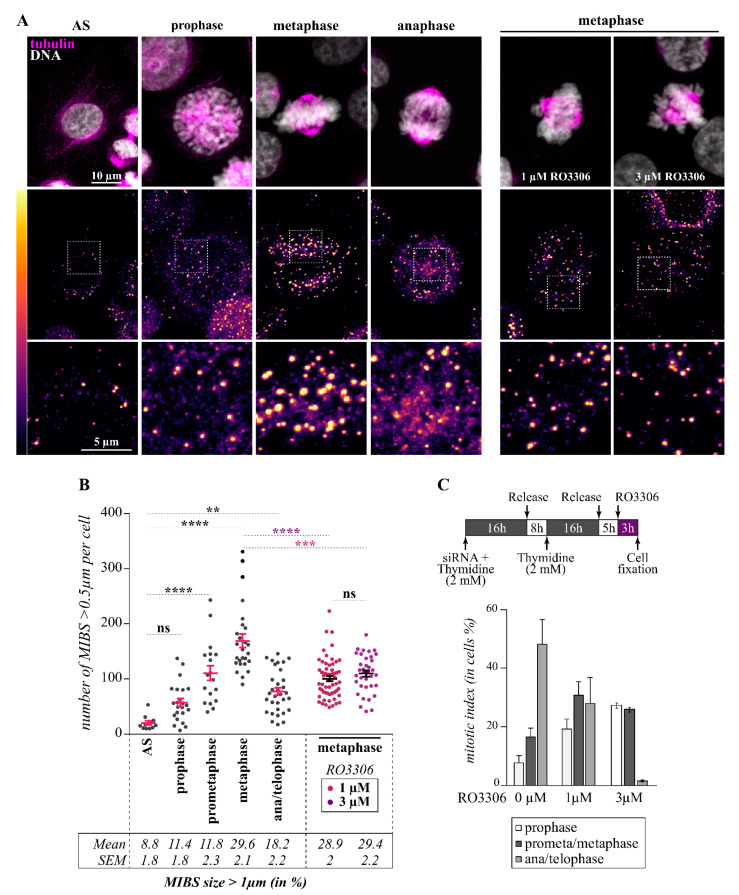
MIBS assembly is regulated by CDK1 activity. (**A**) Maximum intensity projections of confocal image stacks from representative HeLa-RFP-H2B cells synchronized in mitosis by a double thymidine block, showing staining of p62, β-tubulin, and DNA (Hoechst); cells were treated or not with low doses of the CDK1 inhibitor RO3306 for 3 h before cell fixation. Enlarged views of boxed regions are shown to emphasize MIBS assembly until metaphase and their disassembly at anaphase, and inhibition of MIBS assembly by the CDK1 inhibitor. A heatmap pseudo-color intensity scale was applied to the p62 staining channel to underscore MIBS assembly dynamics; Bars: 10 µm or 5 µm**.** (**B**) Quantification of cells from (A), indicating MIBS number per cell and their mean size during mitotic progression, means ± SE from 11 to 59 cells from 2 representative experiments. Statistical significance was analyzed with the Kruskal–Wallis test: ****, *p* < 0.0001; ***, *p* < 0.001; **: *p* < 0.005; ns, not significant. (**C**) Schematic of the protocol used. Quantification of cells from (A), showing percentages of cells treated with RO3306 or the vehicle (DMSO) at distinct mitotic stages, means ± SE from 411 to 438 cells from 2 representative experiments.

Live-cell imaging of inducible Flp-In T-REx HeLa cells expressing p62-RFP further revealed the presence of MIBS in rounded mitotic cells that appeared to disassemble during the progression through cytokinesis (Figure S5C). Thus, we next sought to analyze MIBS assembly during mitotic progression and the impact of CDK1 activity by confocal imaging of p62 immunostained cells to discriminate the mitotic stages by Hoechst staining. We observed that MIBS number and size increased from prophase to metaphase but decreased during anaphase, suggesting that MIBS are dynamics (Figure 6A,B). To determine the role of CDK1 in MIBS assembly, cells were treated with low doses of the CDK1 inhibitor RO3306 during their release from a thymidine block. While 1- to 3 µM RO3306 did not inhibit the mitotic index, cells were delayed in mitosis and accumulated at prometaphase-metaphase, consistent with a partial inhibition of CDK1 (Figure 6C) [74]. Under these conditions, cells at the metaphase stage exhibited a reduction in MIBS number (Figure 6A,B, RO3306). These results suggest that MIBS are dynamic cellular inclusions that are regulated by CDK1.

**Figure 7:**
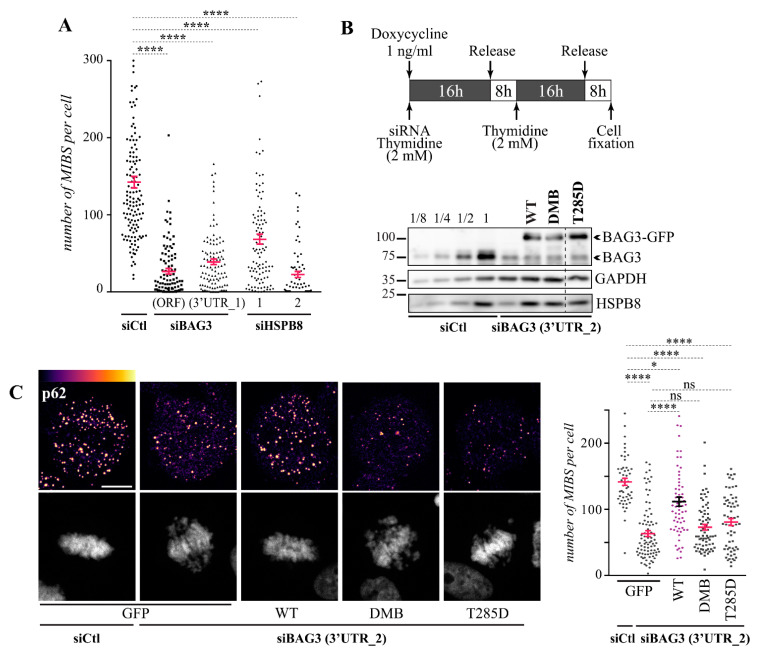
The molecular assembly of p62-MIBS depends on BAG3 phosphorylation and HSPB8. (**A**) Quantification of MIBS number per cell (prometaphase-metaphase) in HeLa cells transfected with control siRNA (siCtl) or BAG3-specific siRNAs or HSPB8-specific siRNAs, and stained for endogenous p62, α-tubulin, and DNA; means ± SE from 70 to 136 cells from at least 3 independent experiments. Statistical significance was analyzed with the Kruskal–Wallis test and Dunn′s multiple comparisons: ****, *p* < 0,0001. (**B**) Schematic of the protocol used for depletion-rescue analyses of MIBS assembly in Flp-In T-Rex HeLa cell lines. Western blots of extracts from cells treated with BAG3-specific siRNA (3′UTR_2) and doxycycline (1 ng/mL, 16 h), showing levels of BAG3-GFP^WT^, BAG3-GFP^DMB^, and BAG3-GFP^T285D^ as compared to endogenous BAG3 levels, as estimated by loading increasing amounts of control Flp-In T-Rex HeLa cell extract (1, 1/4, 1/2, 1/8); HSPB8 and GAPDH levels (loading controls) are shown. (**C**) Maximum intensity projections of confocal image stacks from mitotic Flp-In T-Rex HeLa cell lines expressing GFP as a control or the indicated BAG3-GFP constructs; cells were treated as in (B) and stained for p62, DNA (Hoechst), and β-tubulin (spindle, not shown). A heatmap pseudo-color intensity scale was applied to the p62 staining channel to emphasize impacts on MIBS number and size; Bar: 10 μm. Cells were synchronized in mitosis with a double thymidine block and BAG3-GFP proteins were induced with doxycycline (1 ng/mL, 16 h) during the first thymidine block. The graph shows MIBS number per cell, as quantified in cells at prometaphase-metaphase, means ± SE from 50 to 86 cells from 2 representative experiments. Statistical significance was analyzed with the Kruskal–Wallis test: ****, *p* < 0.0001; *, *p* < 0.05.

Finally, we sought to determine the role of BAG3 and HSPB8 in MIBS assembly. We reasoned that similar to its role during stress, the BAG3-HSPB8 chaperone complex could facilitate p62 molecular assembly [34]. HeLa cells were treated with BAG3- or HSPB8-specific siRNAs and the efficiency of MIBS assembly was monitored by quantification of MIBS number in cells at prometaphase-metaphase in non-synchronized cell populations. We found that depletion of either BAG3 or HSPB8 significantly inhibited MIBS assembly, reducing MIBS number by ~2- to 6-folds (Figure 7A). To then assess the role of BAG3 mitotic phosphorylation, we engineered inducible Flp-In T-REx HeLa cell lines expressing near-to-endogenous levels of BAG3-GFP proteins, including BAG3-GFP^WT^, non-phosphorylatable BAG3-GFP^DBM^, and phosphomimetic BAG3-GFP^T285D^. This approach enabled us to avoid BAG3 overexpression that can promote inclusion formation [36] (Figure 7B). We performed depletion-rescue experiments using siRNA duplexes that target the 3′ untranslated region (3′-UTR) of BAG3. MIBS number were monitored by p62 immunolabeling in single cells expressing similar levels of each BAG3-GFP protein. We found that BAG3-GFP^WT^ expression but not GFP could normalize MIBS number in BAG3-depleted cells. However, neither the non-phosphorylatable BAG3-GFP^DMB^ nor phosphomimetic BAG3-GFP^T285D^ could rescue MIBS assembly, despite that they were able to restore HSPB8 levels in BAG3-depleted cells (Figure 7B,C). We concluded that BAG3 phosphorylation and HSPB8 regulate MIBS assembly during mitosis.

## 4. Discussion

In this study, we identify BAG3 as a *bona fide* substrate of CDK1 that collaborates with its chaperone partner HSPB8 to regulate the molecular assembly of p62 into high-ordered mitotic inclusion bodies (MIBS). Since BAG3, HSPB8 and p62 show mitotically regulated associations and can recapitulate the same mitotic phenotypes, we speculate that MIBS may provide a platform for the fine-tuning of multiprotein complex assembly and/or cytoskeletal component organization that regulate mitotic cell shape changes.

Our first analysis of MIBS assembly suggests that they represent specific ubiquitin-controlled sorting centers that respond to mitotic signals. The sequestosome p62 can form droplets and undergoes phase separation, a process that is promoted by its association with K63 polyubiquitin chains [72]. Moreover, p62 phase separation can be regulated by phosphorylation. Here, we found that MIBS are dynamic spherical structures regulated by CDK1 that are enriched in CDK1-phosphorylated p62 and K63 polyubiquitin chains. These properties, including a labile association with K63 polyubiquitin chains, are consistent with current models for the organization of specific p62 sequestering structures [60,72,75]. By condensing biomolecules, MIBS could facilitate the timed changes in mitotic cytoskeletal organization. Compartmentalized signaling through biomolecular condensates emerges as a key process to alter the kinetics and specificity of biochemical reactions and sequester molecules [76,77]. In line with this possibility, we found that p62 depletion markedly affected CDK1-mediated BAG3 phosphorylation. Thus, p62-MIBS could fine-tune CDK1-mediated phosphorylation events by concentrating or sequestering protein substrates. Alternatively, MIBS could provide a platform to adjust HDAC6-mediated deacetylation of specific substrates. In line with this possibility, recruitment of HDAC6 to the BAG3-HSPB8-p62 complex during mitosis is associated with a decreased HDAC6-cortactin association that correlates with an increase cortactin acetylation [6,18]. Thus p62-MIBS could contribute to the rapid tuning of branched actin polymerization, a process that is regulated by BAG3, HSPB8, and p62 [18,19]. While MIBS remain to be deciphered at a functional level, they support the existence of significant connections between protein quality control (PQC) and mitotic signaling. To our knowledge, this is the first report of a mitotically regulated change in the supramolecular organization of p62 that clearly warrants further investigations that are beyond the scope of the current study.

Our findings also argue that dynamic cycles of BAG3 phosphorylation-dephosphorylation at the BAG3 S284-T285 motif may regulate the activity of the BAG3-HSPB8 chaperone complex during mitosis. Indeed, CDK1 pharmacological inhibition during early mitosis impaired BAG3 phosphorylation. Moreover, the non-phosphorylatable BAG3 and phosphomimetic BAG3 protein both displayed a severe loss-of-function during mitosis. Based on MS data, T285 may be the preferred phospho-site during mitosis, although S284 was also phosphorylated in a mitosis-specific manner. Moreover, pT285 was validated as a mitotic phospho-site using various approaches. Since we did not examine the individual contribution of S284 and T285 to BAG3 mitotic function, their relative role remains unclear. CDK1-dependent regulation relies on cumulative phosphorylation of individual proteins, as well as multiple proteins within defined signaling pathways [55]. In line with this concept, both p62 [59] and BAG3 (this study) contain two or more mitotically regulated phospho-sites. We think unlikely that a mutagenesis approach could enable discriminating the functional contribution of neighbor residues on BAG3, as this might require the use of quantitative phosphoproteomics. However, mutation of T285 to an Asp was sufficient to impair BAG3 mitotic function, validating a requirement for T285 mitotic regulation. Most importantly, the same constructs retain some of BAG3′s functions, notably their ability to stabilize HSPB8 levels that depends on a stable interaction with BAG3. Thus, we infer that these BAG3 construct loss-of-function during mitosis is not due to unspecific structural defects [19,25,34]. The reason underlying a requirement for BAG3 phosphorylation dynamic is currently not known. However, it is interesting to speculate on how it may contribute to MIBS assembly. We found that p62 biochemical interactions with both the BAG3 cochaperone and polyubiquitin chains during mitosis are labile. The predicted mode of chaperoning by HSPB proteins involves cycles of binding and release of substrate proteins [78]. Thus, changes in the BAG3 phosphorylation state, which are likely to modify BAG3-HSPB8 complex structure, could facilitate the recruitment of K63 polyubiquitin-tagged proteins to promote MIBS assembly, followed by the subsequent release of mature MIBS. However, our unpublished data suggest that site-specific BAG3 phosphorylation could have complex effects on BAG3 partnerships, which will require to be dissected using in vitro reconstitution systems. Apart from the hereby described phosphosites, we identified others CDK1-targeted phospho-motifs on BAG3, notably within the BAG3 PXXP motif that is essential for BAG3 mitotic function.

In conclusion, this work provides significant mechanistic advances on the mitotic BAG3-HSPB8-p62 signaling axis, by uncovering a requirement for CDK1-mediated BAG3 phosphorylation and the mitotically regulated assembly of a p62 bodies: MIBS. Deciphering MIBS at molecular and functional levels should provide fundamental insights into the biology of mitotic progression.

## Data Availability

All data that support the findings of this study are available from the corresponding author (josee.lavoie@crchudequebec.ulaval.ca) upon reasonable request.

## Supplementary Materials

The following are available online at https://www.mdpi.com/article/10.3390/cells10102638/s1. Figure S1: Analysis of BAG3 phosphorylated residues by AP-MS. (A) BAG3 IPs were prepared using anti-BAG3 from asynchronous (AS) HEK 293T cells or from cells synchronized in mitosis by nocodazole treatment (400 ng/mL, 16 h) and recovered by mitotic shake-off (M). BAG3 bands were revealed by Coomassie staining after SDS-PAGE and bands delineated by boxed regions were excised and submitted to MS/MS analysis of the phosphopeptide content after in-gel digestion; blue box: interphase BAG3 band; red box: mitotic supershifted BAG3 band. (B) BAG3 phospho-sites were determined from 6 independent biological replicates and the relative extent of amino acid phosphorylation is expressed as the number of replicates in which phosphorylation was detected relative to the number of replicates showing coverage of the residue; pS284 and pT285 were detected only in the mitotic BAG3 supershifted band (in boldface) (C-F) Representative MS/MS spectra for phosphopeptides containing the following residue: S289 (A), S291 (B), T285 (C) and S284 (D). Mascot scores for peptide identification, and Ascores for the localization of phosphorylated sites, are indicated. The total number of peptides for each residue are as follows: pS284: 2; pT285: 13; pS289:33; and pS291:14; from 6 independent experiments; m/z, mass per charge; AMU, atomic mass unit. Figure S2: (A) Western blots of total cell extracts from HeLa-RFP-H2B cells showing the relative efficiency of BAG3 depletion upon transfection of siRNA sequences used in this study; levels of endogenous BAG3 depletion were estimated by loading increasing amounts of control cell extract (siCtl, 1/8, 1/4, 1/2, 1). (B) Western blots of extracts from mitotic HeLa-RFP-H2B (nocodazole-arrested) that have been treated with the CDK1 inhibitor RO3306 for various periods of time (8 µM for 0–60 min), using anti-PY15-CDK1 (inactive CDK1), anti-CDK1, anti-pT285-BAG3, and anti-BAG3; vinculin levels are shown as a loading control. Mitotic cells were recovered by mitotic shake-off; representative of 2 independent experiments. (C) Western blots of extracts from mitotic HeLa-RFP-H2B (nocodazole-arrested) that have been pretreated with the proteasome inhibitor MG132 (5 µM for 30 min) before adding the CDK1 inhibitor RO3306 (8 µM for 30 min), showing BAG3 mitotic mobility shift (anti-BAG3) and cyclin B1 levels when mitotic exit is blocked; GAPDH levels are shown as a loading control. Figure S3: Non-phosphorylatable BAG3-GFP^DMB^ expression in BAG3-depleted cells can rescue aggresome formation. (A) Schematic of the protocol used to perform depletion-rescue experiments. Cells were treated with a BAG3-specific siARN targeting the 3′UTR region (siBAG3 [3′UTR_1]), transduced with Ad-GFP or Ad-BAG3-GFP as indicated, and then treated with 5 µM of MG132 for 16 h. Cells were fixed and processed for immunostaining of endogenous p62; DNA was stained with Hoechst. (B) Maximum intensity projections from confocal image stacks showing dispersed microaggregates of p62 (siBAG3 + GFP) or a perinuclear aggresome (white arrows: siCtl + GFP, siBAG3 + WT, siBAG3 + DMB). (C) The graph depicts percentages of cells with p62 dispersed aggregates versus a perinuclear aggresome; means ± SE from 394 to 926 cells from 2 to 4 independent experiments. Figure S4: (A) BAG3-GFP IPs were prepared from mitotic HeLa-RFP-H2B cells transduced with recombinant adenoviruses driving the expression of wild-type (WT) BAG3-GFP or BAG3-GFP constructs defective in HSPB8 binding (IPV) or bearing a deletion of the PXXP motif. Cells were synchronized in mitosis by nocodazole and collected by mitotic shake-off. Immunoblotting was performed using anti-p62, anti-HSPB8, and anti-GFP antibodies. Levels of CDK1, p62, HSPB8, and GFP or BAG3-GFP in total cell extracts are shown (Input). (B) Graph depicting the fold change in total BAG3 levels upon p62 depletion, as estimated relative to cells transfected with control siRNA and normalized relative to GAPDH levels; means ± SE from 4 independent experiments. Statistical analysis by the Kruskal–Wallis test: not significant (ns). Figure S5: (A) Depletion of p62 or HSPB8 impairs MIBS formation. Maximum intensity projections of confocal image stacks of representative HeLa Flp-In TREx cells at the metaphase stage showing a decrease in p62-MIBS; a heatmap pseudo-color intensity scale was applied to the p62 channel to emphasize the decrease in p62 condensates; DNA was stained with Hoechst. Bar: 10 µm. (B) Maximum intensity projections of confocal image stacks of asynchronous mouse myoblasts (C2C12 cells) or HeLa CCL-2 cells (American Type Culture Collection) showing an increase in p62 clusters in cells at the metaphase stage; staining of mitotic p62 phosphorylated at CDK1-target sites (pT269-S272) and DNA (Hoechst) are shown as indicated. Bar: 20 µm. (C) Representative spinning disk confocal time-lapse sequences of two Flp-In T-REx HeLa cells expressing p62-RFP, showing MIBS dynamics during mitotic progression. Cells were treated with doxycycline (10 ng/mL for 16 h) in the presence of the CDK1 inhibitor to arrest cells at the G2/M border (RO 3306, 4 µM). Cells were then washed to release them in mitosis and imaged for 1 h at 30 sec intervals using a Perkin Elmer UltraVIEW Spinning Disk Confocal and a 40 × air 0.75 NA objective. A heatmap pseudo-color intensity scale was applied to the p62 channel to emphasize changes in the intensity and size of p62 clusters (MIBS) during mitotic progression. Video S1: related to Figure 3C. Video microscopy of a representative rounded HeLa-RFP-H2B cell transfected with control siRNA (siCtl) and expressing low levels of RFP-α-tubulin; the white asterisk designates one spindle pole showing a normal positioning dynamic. Mitotic cells were imaged for 75 min at ~1.5 min intervals using a Perkin Elmer UltraVIEW Spinning Disk Confocal and a 40 × air 0.75 NA objective; single plane images are displayed at 2 frames/sec. Video S2: related to Figure 3C. Video microscopy of a representative rounded HeLa-RFP-H2B cell treated with BAG3-specific siRNA (siBAG3 [3′UTR_1]) expressing low levels of RFP-α-tubulin; the cell shows a typical spindle orientation defect that is manifested by spindle rocking, as reflected by the unusual motility of the spindle pole designated by the white asterisk. Mitotic cells were imaged for a 75 min-period at ~1.5 min intervals using a Perkin Elmer UltraVIEW Spinning Disk Confocal and a 40 × air 0.75 NA objective; single plane images are displayed at 2 frames/s. Video S3: related to Figure 3C. Video microscopy of a representative rounded HeLa-RFP-H2B cell transfected with BAG3-specific siRNA (siBAG3 [3′UTR_1]) and cotransduced with Ad-BAG3-GFP^WT^ and BacMAm.RFP-α-tubulin, showing normalized mitotic spindle dynamics; the white asterisk designates one spindle pole showing a rapid positioning. Mitotic cells were imaged for a 75 min-period at ~1.5 min intervals using a Perkin Elmer UltraVIEW Spinning Disk Confocal and a 40 × air 0.75 NA objective; single plane images are displayed at 2 frames/sec. Video S4: related to Figure 3C. Video microscopy of a representative rounded HeLa-RFP-H2B cell transfected with BAG3-specific siRNA (siBAG3 [3′UTR_1]) and cotransduced with Ad-BAG3-GFP^DMB^ and BacMam.RFP-α-tubulin that shows spindle rocking, as reflected by the unusual motility of the spindle pole designated by the white asterisk. Mitotic cells were imaged for a 75 min-period at ~1.5 min intervals using a Perkin Elmer UltraVIEW Spinning Disk Confocal and a 40 × air 0.75 NA objective; single plane images are displayed at 2 frames/sec. Video S5: related to Figure 3C. Video microscopy of a representative rounded HeLa-RFP-H2B cell treated with BAG3-specific siRNA (siBAG3 [3′UTR_1]) that expresses BAG3-GFP^T285D^ and low levels of RFP-α-tubulin that shows mitotic spindle rocking, as reflected by the unusual motility of the spindle pole designated by the white asterisk. Mitotic cells were imaged for a 75 min-period at ~1.5 min intervals using a Perkin Elmer UltraVIEW Spinning Disk Confocal and a 40 × air 0.75 NA objective; single plane images are displayed at 2 frames/s.

## Supplemental Methods: Mass Spectrometry and Phospho-Peptides Analyses

HEK 293 T were treated or not with nocodazole (400 ng/mL 16 h), collected using PBS supplemented with 15 mM citrate and 0.6 mM EDTA, and lysed in 10 volumes of modified RIPA buffer (TRIS-HCl 50 mM pH 7,6, 150 mM NaCl, 1 mM EDTA, 1% Igepal, 1X complete w/o EDTA [Roche], 10 mM NaF, 1 mM sodium orthovanadate, 40 mM β-glycerophosphate). After centrifugation at 15,000 xg for 15 min, supernatants were incubated for 60 min with an anti-myc antibody. Immune complexes were absorbed on Dynabeads-protein G (Invitrogen) for 30 min, washed on a magnet 3 times with RIPA buffer, and briefly separated on SDS-PAGE. BAG3 protein bands were revealed with Coomassie Blue R250 and excised for protein-in-gel digestion. The bands of interest from SDS-PAGE were placed in 96-well plates and then washed with water. Tryptic digestion was performed on a MassPrep liquid handling robot (Waters) according to standard methods [79], with modifications as suggested [80]. Briefly, proteins were reduced with 10 mM DTT and alkylated with 55 mM iodoacetamide. Trypsin digestion was performed using 126 nM of modified porcine trypsin (Sequencing grade, Promega) at 58 °C for 1 h. Digestion products were extracted using 1% formic acid, 2% acetonitrile followed by 1% formic acid, 50% acetonitrile. The recovered extracts were pooled, vacuum centrifuge dried and then resuspended into 10 µL of 0.1% formic acid, and 2 µL were analyzed by mass spectrometry. In-gel digestion and mass spectrometry were performed at the proteomics platform of the CRCHU de Québec-Université Laval, Qc, Canada. Peptide samples were separated on a TripleTOF 5600 mass spectrometer fitted with a nanospray III ion source (ABSciex) and coupled to an Agilent 1200 HPLC. The sample was injected onto a self-packed 0.075 mm x 15 cm, 5 u, C18 Jupiter (Phenomenex) PicoFrit column (New Objective). Samples were run using a 65 min gradient from 5–35% solvent B (solvent A 0.1% formic acid in water; solvent B: 0.1% formic acid in acetonitrile) at a flow rate of 300 nl/min. The mgf files were generated using Protein Pilot version 4.5 software (Sciex) and analyzed using Mascot (Matrix Science, version 2.4.0) and Tandem (The GPM, thegpm.org; version CYCLONE (2010.12.01.1). Mascot was set up to search the TAX-HomoSapiens-9606 (November 2014) database. Mascot was searched with a fragment ion mass tolerance of 0.10 Da and a parent ion tolerance of 0.10 Da. For BAG3 phosphorylated amino acid determination, carbamidomethyl of cysteine was specified in Mascot as a fixed modification, dehydrated of the n-terminus, glu- > pyro-Glu of the n-terminus, ammonia-loss of the n-terminus, gln- > pyro-Glu of the n-terminus, deamidated of asparagine and glutamine, oxidation of methionine and phosphorylation of serine, threonine and tyrosine were specified as variables modifications. Glu- > pyro-Glu of the n-terminus, gln- > pyro-Glu of the n-terminus, deamidated of asparagine and glutamine, oxidation of methionine and phosphorylation of serine, threonine and tyrosine were specified in Mascot as variable modifications. Two missed cleavages were allowed. Scaffold PTM (Proteome Software, Portland, Oregon, USA) was used to annotate Post Translational Modification sites. The program utilizes the ASCORE’s probabilistic approach and scoring technique [52].
