# Supplementary figures and images for: CDK1-Mediated Phosphorylation of BAG3 Promotes Mitotic Cell Shape Remodeling and the Molecular Assembly of Mitotic p62 Bodies
Source: Cells. 2021 Oct 2;10(10):2638. doi: 10.3390/cells10102638 (PMC8534064; doi:10.3390/cells10102638)

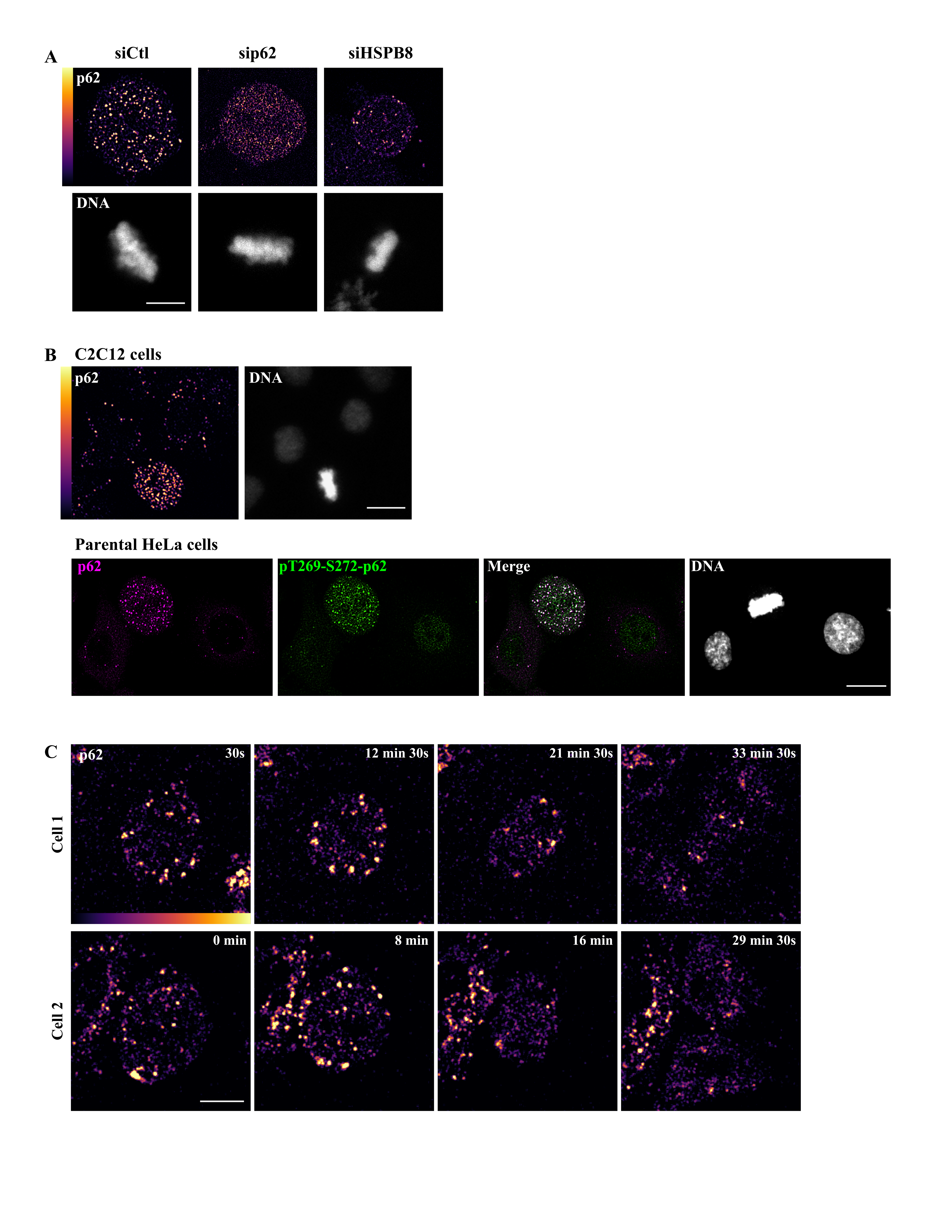

Supplement: Supplementary file 1 [file cells-10-02638-s001.zip › Supplementary materials/figS5R.tif]

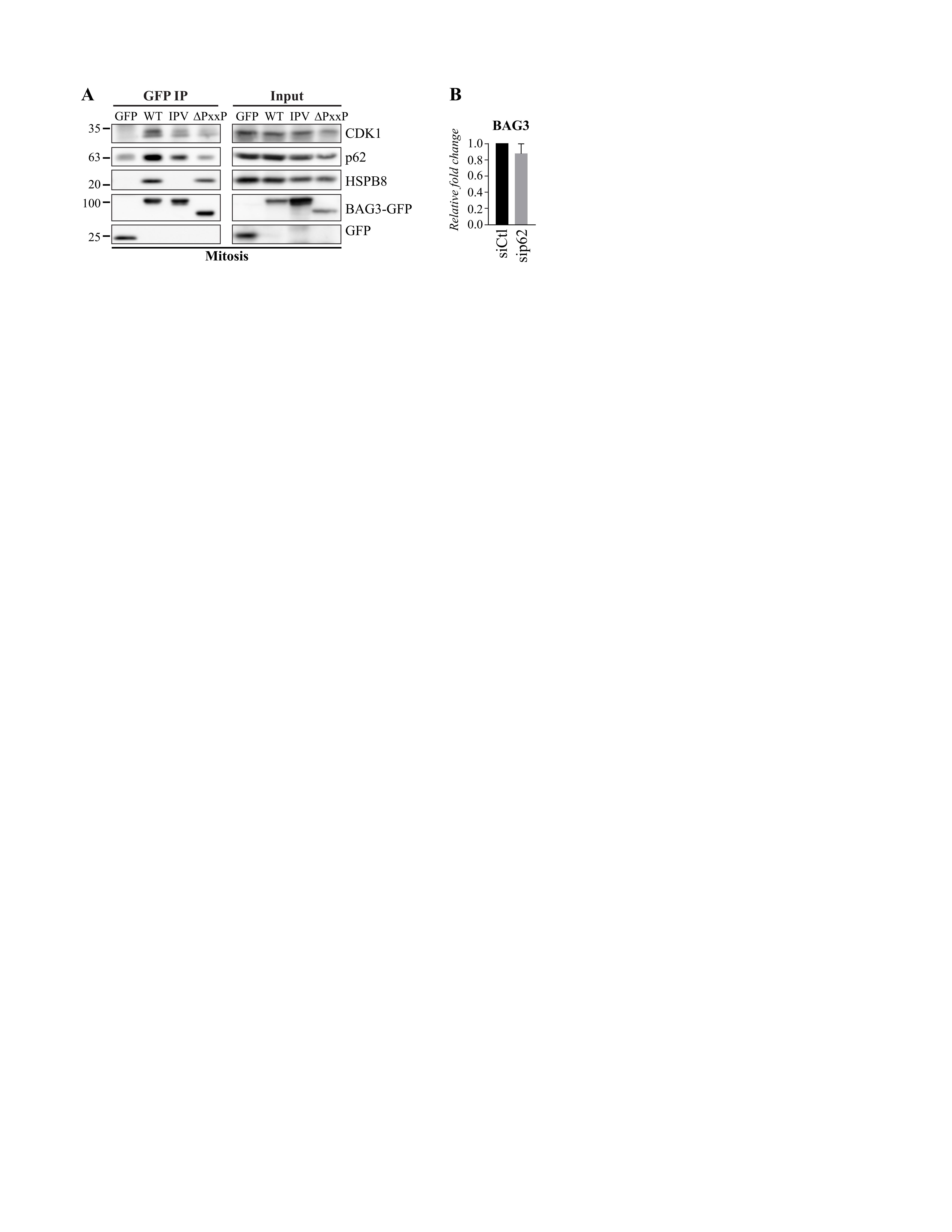

Supplement: Supplementary file 1 [file cells-10-02638-s001.zip › Supplementary materials/FigS4R.tif]

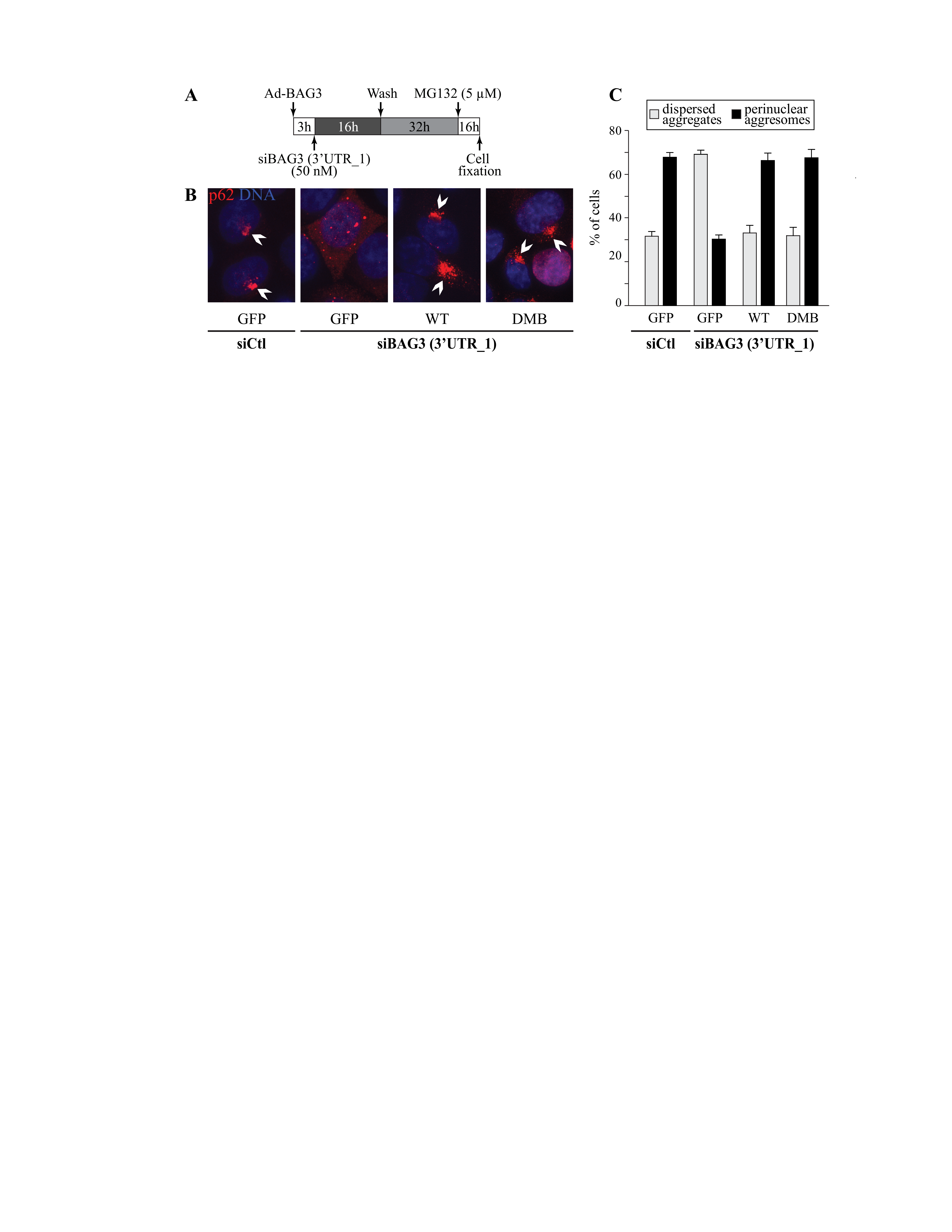

Supplement: Supplementary file 1 [file cells-10-02638-s001.zip › Supplementary materials/figS3R.tif]

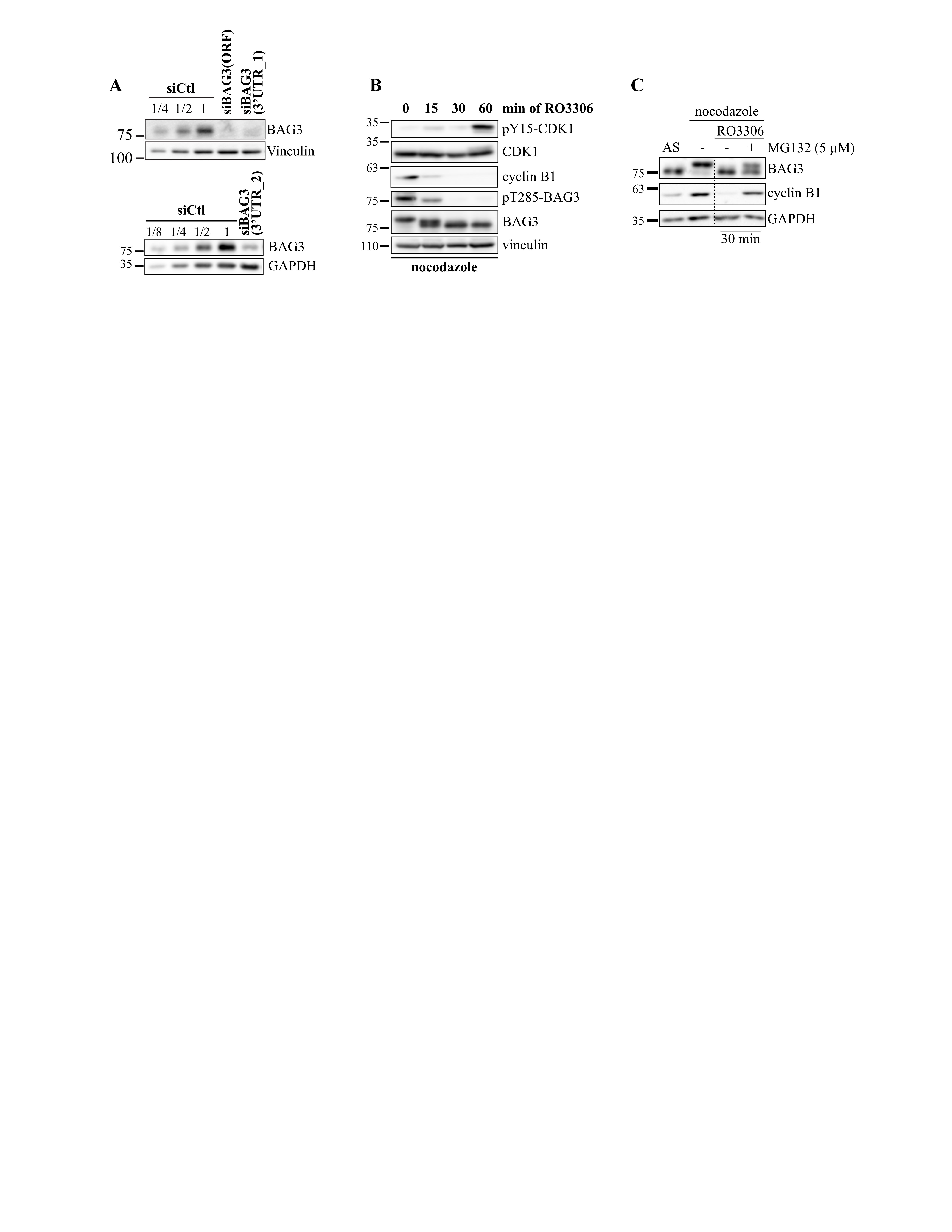

Supplement: Supplementary file 1 [file cells-10-02638-s001.zip › Supplementary materials/FigS2R.tif]

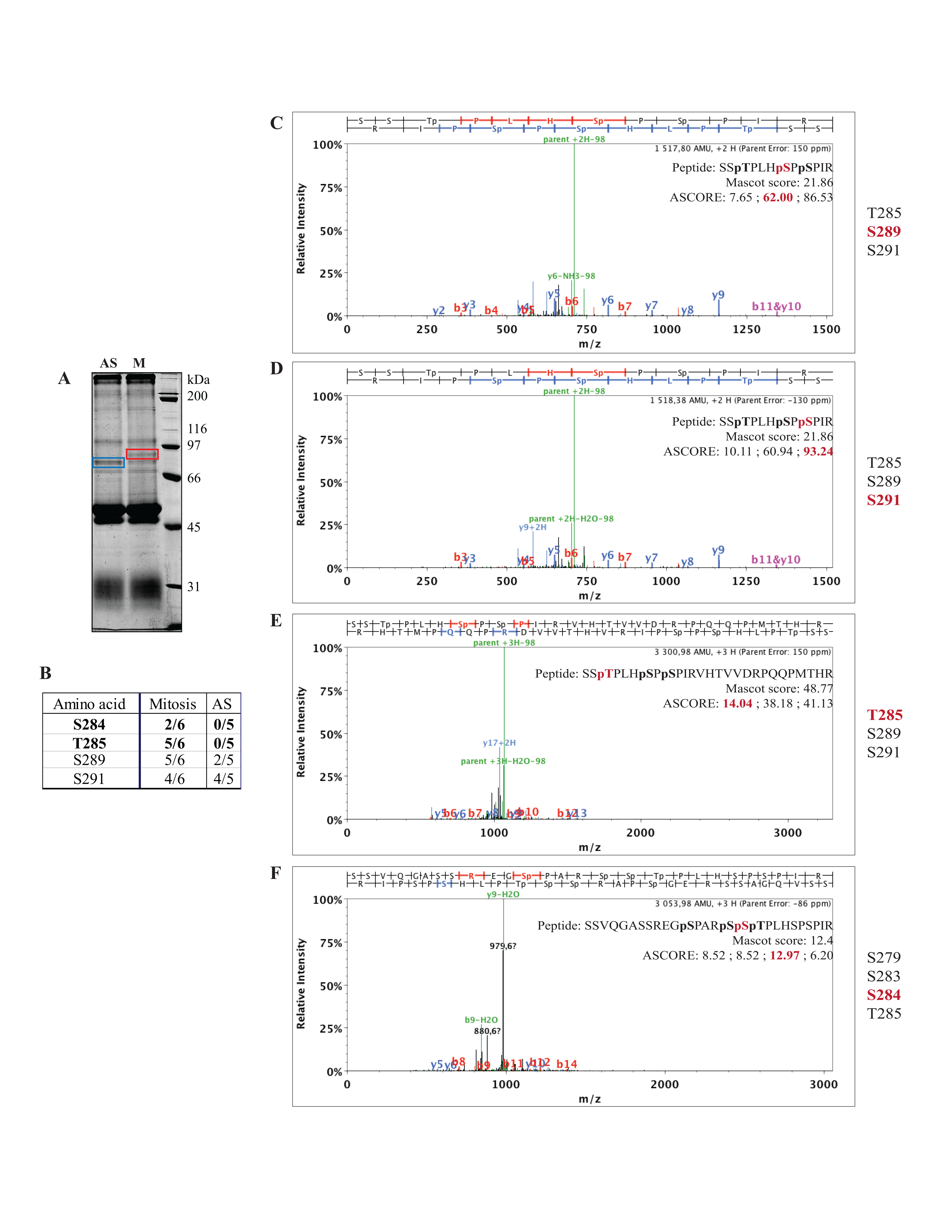

Supplement: Supplementary file 1 [file cells-10-02638-s001.zip › Supplementary materials/FigS1R.tif]
